# Morphological and acoustic characters of *Cicadatra platyptera* Fieber, 1876

**DOI:** 10.3897/zookeys.296.4855

**Published:** 2013-04-25

**Authors:** Abbas Mol, Unal Zeybekoglu, Basak Akyurek

**Affiliations:** 1 Aksaray University, Guzelyurt Vocational School, Guzelyurt, Aksaray, Turkey; 2 Ondokuz Mayis University, Science and Art Faculty, Biology Department, Samsun, Turkey

**Keywords:** Hemiptera, Cicadidae, *Cicadatra platyptera*, *Cicadatra atra*, morphology, acoustics, Turkey

## Abstract

Acoustic and morphological characters are very important to distinguish species of Cicadidae. In this study, the morphological and acoustic characters of *Cicadatra platyptera* Fieber, 1876 (Hemiptera, Cicadidae) collected from Turkey were analysed. The external morphological structures of two species were drawn and photographs of some specimens were taken. We evaluated taxonomically important morphological characters such as body shape, colors, patterns, structure, and genital structure. We evaluated measurements of external morphological structures and acoustics characters of *Cicadatra platyptera* from Turkey, partly with statistical analyses. Morphological characters were compared and differentiated from the closely related species, *Cicadatra atra*. The distribution in Turkey including previous records and the material examined were shown on a map, and the distribution in Palearctic Region was given.

## Introduction

It is known that there are more than 40 species of the genus *Cicadatra* Kolenati, 1857 many of which are distributed in Middle East countries and surrounding areas (Nast 1972, Duffels and Laan 1985, Boulard 1995, Gogala and Trilar 1998, Schedl 1999, Mozaffarian and Sanborn 2010, Mozaffarian et al. 2010, Ahmed et al. 2012, Simoes et al. 2012) including Anatolia. Nast (1972), Claridge (1985), Duffels and Laan (1985), Schedl (1999), Gogala and Trilar (1998, 2003), Gogala et al. (2005), and Simões et al. (2012) have studied morphological characters of *Cicadatra*taxa and also the acoustic characters of some species in the Palearctic Region.

Nast (1972), Kartal (1980), Lodos and Kalkandelen (1981), Koçak and Kemal (2010), and Boulard (1995) listed 10 species in genus *Cicadatra* Kolenati, 1857 from Turkey. These species are *Cicadatra alhageos* (Kolenati, 1857); *Cicadatra atra* (Olivier, 1790); *Cicadatra adanai* Kartal, 1980; *Cicadatra platyptera* Fieber, 1876; *Cicadatra hyaline* (Fabricius, 1798); *Cicadatra tenebrosa* Fieber, 1876; *Cicadatra querula* (Pallas, 1773); *Cicadatra persica* Kirkaldy, 1909, *Cicadatra glycirrhizae* (Kolenati, 1857), and *Cicadatra hagenica* Dlabola, 1987. Three of them are endemic for Turkey. These are *Cicadatra adanai*, *Cicadatra tenebrosa*, and *Cicadatra glycirrhizae* (Duffels and Laan 1985, Boulard 1995).

Acoustic signals in insects are widely used both for intra- and inter-specific communication. The loud airborne sounds of many groups of large cicadas are well known (Claridge 1985, Quartau et al. 1999, Simões et al. 2000, Fonseca and Revez 2002, Quartau and Simoes 2006, Mozaffarian and Sanborn 2010). In cicadas, the sound production apparatus is known as a timbal mechanism and has a versatile system able to produce several kinds of sound signals in different behavioral contexts (Claridge 1985, Fonseca 1991). Claridge (1985) reported that the acoustic behavior of Auchenorrhyncha is considered under the following simplified series of categories after Alexander: (a) disturbance and alarm, (b) calling, (c) aggression, (d) courtship and copulation.

Different acoustic parameters of continuous song, calling song, courtship song, and alarm song are very important to determine relationships between some Auchenorrhyncha species (Claridge 1985, Fonseca 1991, Gogala and Trilar 2004, Moore 1993, Simões et al. 2000, Zeybekoğlu et al. 2011).

Nast (1972), Kartal (1980), Lodos and Kalkandelen (1981), and Kocak and Kemal (2010) had studied the morphological characters of *Cicadatra* species in Turkey. In addition, there are a few sound records of Anatolian populations of the species in the genus (Boulard 1995). However, it seems that there are still very little data on *Cicadatra* species in Turkey, specifically there are not any thorough studies about their morphological and behavioral characters. One of these species is *Cicadatra platyptera* Fieber, 1876. The current study is an investigation on the morphological and acoustic characters of *Cicadatra platyptera* species collected from different localities of Turkey. Until now, Boulard (1995) and Gogala et al. (2005) studied some song characters of *Cicadatra platyptera*, but they did not study their alarm song.

Thus, in the present study, we aimed (i) to review *Cicadatra platyptera* Fieber, 1876 thoroughly using morphological characters; (ii) to describe calling songs with variations, courtship song and alarm song; (iii) to evaluate their relationship with the closely related species in terms of some morphological and acoustics characters.

## Methods

The research materials were *Cicadatra platyptera* (Cicadidae) adult specimens collected from Turkey (Fig. 17). Firstly, the sounds of adult males living in natural habitat were recorded. Field recordings of the songs were done with a Sony Cassette-Recorder WM-GX 688 and with a Sony flat frequency response microphone (50 Hz to 18 kHz). Then, the specimens were collected with a sweep net and prepared as per standard methods. The important taxonomic characters of prepared specimens such as external morphological structures and genital structure were examined and were drawn or photographed with a digital camera or camera lucida attached to a stereo microscope. In order to compare parameters, SPSS (15.0) software package was used and T-test of independent-samples was applied. Differences of P< 0.05 were considered as significant. Male songs which were recorded with Sony Cassette-Recorder were transferred to computer with COOL EDIT 96 software and filtered to clear the sound, then were analysed with TURBOLAB 4.0.Oscillograms and frequency (digitalized at 44100 Hz) of the sound of male calling songs were prepared and analysed by using COOL EDIT 96, TURBOLAB 4.0 (STAMMER AG) and ADOBE PHOTOSHOP programs on computer.

To identify the specimens as *Cicadatra platyptera* Fieber, 1876, we used the morphology by Dantsig et al. (1964), Quartau (1988), Schedl (1999), Moulds (2005) and used acoustic characters by Gogala and Trilar (1998), Gogala et al. (2005), Hertach (2011), and Gogala (2013). All samples are stored in the Aksaray University Central Research Laboratory (ASUBTAM) (Aksaray/Turkey).

## Results

### Family Cicadidae. Genus *Cicadatra*Kolenati, 1857

**Type species.** *Cicadatra atra* (Oliver, 1790)

#### Cicadatra platyptera

Fieber, 1876

http://species-id.net/wiki/Cicadatra_platyptera

Figs 1 [Fig F2] [Fig F3] [Fig F4] [Fig F5] [Fig F6] [Fig F7] [Fig F8] –12 17 [Fig F15] [Fig F16] –21 Table 1 [Table T2] [Table T3] –4

##### Morphology.

*Redescription*: Male. General appearance of body black with yellowish and white pile.

*Head*. General color of head blackish yellow with white scattered pile. Vertex blackish with sparse pile and some specimens have yellowish area between ocelli and top of postclypeus surrounded by yellowish area. Eyes yellowish brown and rarely blackish. Ocelli reddish brown and rarely yellowish. Epicranial suture generally yellowish. Antennae blackish. Frons black with white pile. Postclypeus with a central sulcus obvious transverse grooves with long pile specifically located near gena and edge of mandibular plate, transverse grooves blackish. Gena brownish with white pile specifically below the antennae. Mandibular plate yellowish with white pile. Anteclypeus yellowish brown with blackish area dorsally and white pile on lateral edge. Rostrum almost reaches coxae III, yellowish brown at base, darker towards apex, rarely yellowish laterally and with sparse pile (Fig. 1-A).

**Figure 1-A.**
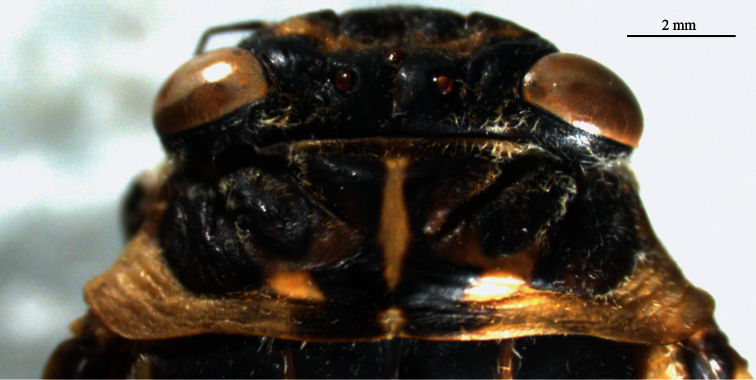
*Cicadatra platyptera*, head and pronotum (scale= 2 mm).

*Thorax*. Pronotum blackish, wider than vertex and twice as long as it, pronotumwith three large patch divided by pronotal and lateral fissure in both half and both fissure with white scattered pile (Fig. 1-A). Both patches brownish black. Lateral angle of pronotal collar widened, ambient fissure nearly smooth. Mesonotum narrowed posteriorly like a bow and blackish with M-shaped yellowish fasciae. Sometimes U-shaped on parapsaidal sutures and nearly cover mesonotum. Lateral part of mesonatal collar and metanotum yellowish with short piles, scutal depression spoonlike and blackish, scutellum yellowish and blackish on both side (Fig. 1-B).

**Figure 1-B. F2:**
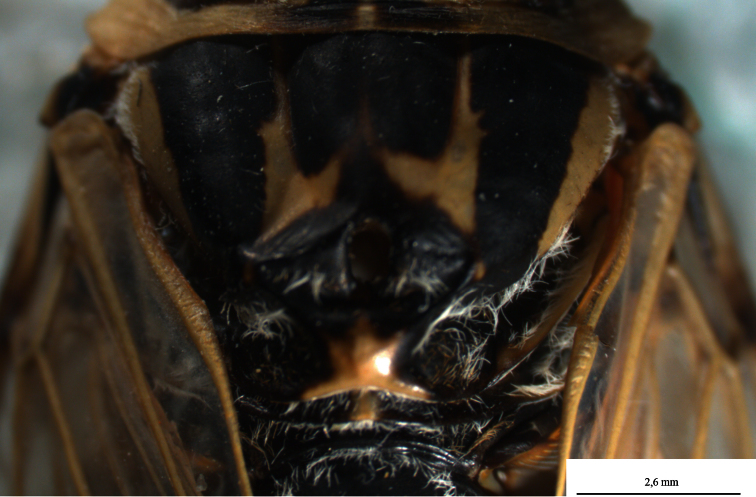
*Cicadatra platyptera*, mesonotum (scale= 2 mm).

*Legs*. Fore coxae rectangle-shaped, with mid-cavity, yellowish with pile and black band basally. Middle and hind coxae trapezoid-shaped, shiny yellowish with pile and middle coxae with a black band basally, hind coxae with half depression in the middle. Fore, middle, and hind trochanters yellowish with dark brown basally, with piles at both corners, sometimes hind trochanters only yellowish. Fore femorae dark brown with white piles and yellow areas on lateral edges, sometimes areas irregular. Slightly angled primary spine, erect secondary spine and nearly erect apical spine, spines and surrounding areas blackish. Middle femorae yellowish with one or two dark brown bands dorsally and white pile. Hind femorae yellowish with white pile and brownish band dorsally. Fore tibia blackish brown with dense pile especially ventrally. Middle tibia yellowish, with piles and varying one to two brownish bands. Hind tibia yellowish with piles and five brown tibial spurs and sparse white pile. Tibial spurs and combs brown, darker distally. Fore tarsi blackish, middle tarsi black-brownish and hind tarsi yellow-brownish. Claws brownish basally and darker distally.

*Wings*. Fore wings at rest roof shaped, covering the abdomen and hyaline with yellowish venation basally and venation apically, R+Sc veins blackish. Basal cell on fore wings heptagonal, cubitus anterior and median veins originate separately. Cubitus posterior and median veins closer at base. Cubitus posterior anal veins not combined at base or apex. Fore wings with 8 apical cells and CuA_1_ wider than other apical cells (Fig. 1-C). Hind wing hyaline with yellowish venation and with 6 apical cells.

**Figure 1-C. F3:**
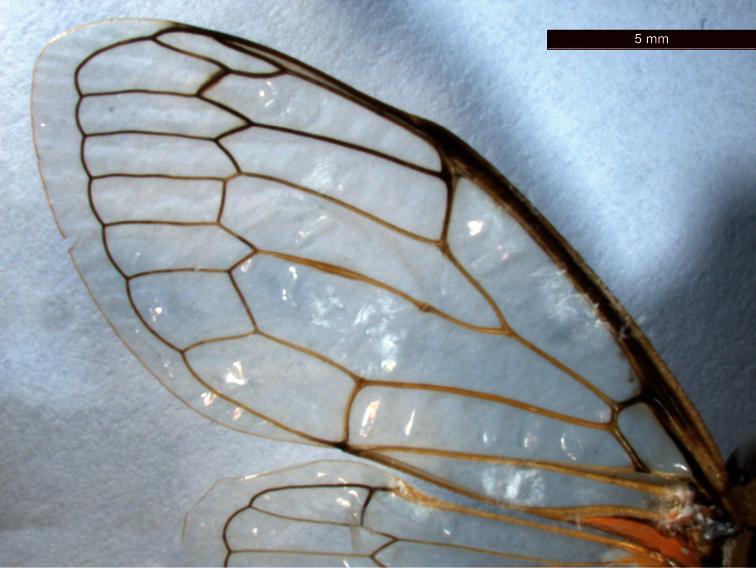
*Cicadatra platyptera*, front fore wing (scale= 2.6 mm).

*Operculum*. Opercula yellow generally with brown spot on lateral base and white pile, broadly rounded apically, approaching one another and meeting medially.

*Abdomen*. Abdominal tergites blackish with white pile more or less located near the anterior edge of each tergum. Generally tergites one to seven with a light area on posterior apical part. Timbal cavity exposed. Timbal cover incomplete, blackish or brownish yellow with white short pile dorsally. Timbal with 11 ribs (Fig. 12). Abdominal sternites yellowish. On sternite II blackish area and on sternite III blackish spot at the base in the middle. Epipleurites yellowish.

**Figure 2. F4:**
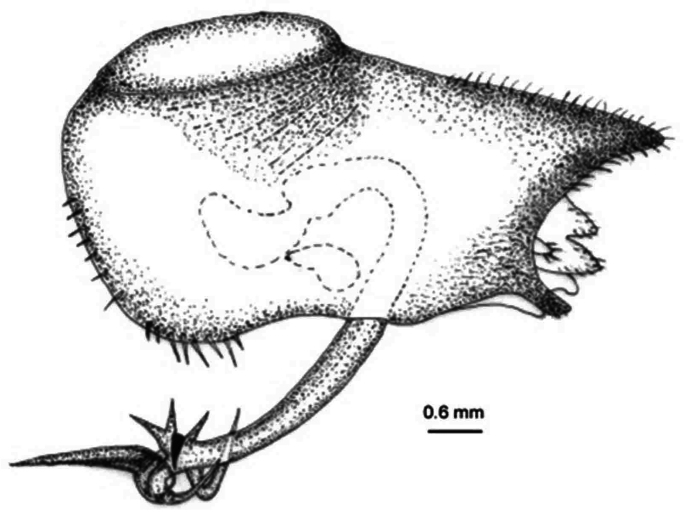
*Cicadatra platyptera*, pygofer (scale= 0.6 mm).

**Figure 3. F5:**
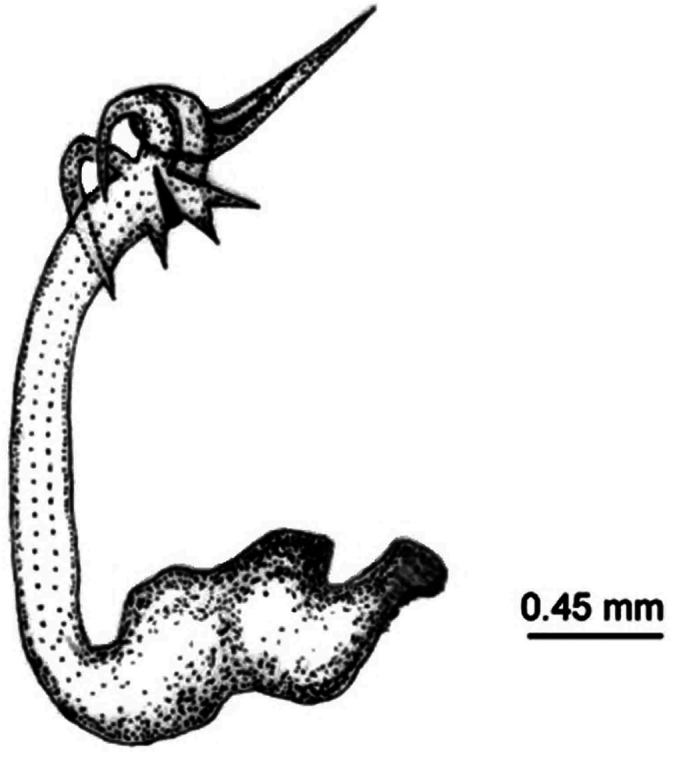
*Cicadatra platyptera*, aedeagus (scale= 0.45 mm).

**Figure 4. F6:**
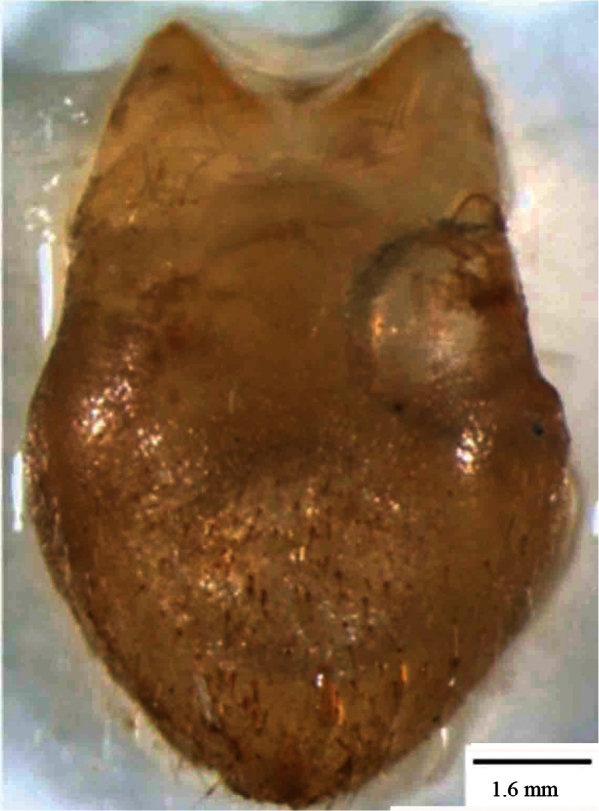
*Cicadatra platyptera*, sternite VIII (scale= 1.6 mm).

*Pygofer*.blackish brown dorsally, yellowish brown ventrally. Posteriorly bow-shaped and sparsely setae dorsa-laterally, smooth dorsally, ventral slightly wavy (Fig. 2). Dorsal beak higher than upper lobe of pygofer and acute. The aedeagus bipartite basally, extending apically in a pipe shape, at apex pointed appendages like lamellae (Fig. 3). Sternite VIII concave basally, widened in the middle and slightly narrows to apex (Fig. 4).

##### Acoustics.

References to song: Boulard (1995), Gogala et al. (2005) and Gogala (2013).

Males of *Cicadatra platyptera* make sounds by using the timbals and fore wings. We were able to record clearly three different types of song, these are: calling song with repeating echemes, produced obviously by timbals like in the close related species *Cicadatra atra*; courtship song produced obviously by a combination of timbale echemes and wing clicks, which is also typical for courtship songs of some other *Cicadatra* species (Boulard 1995); and alarm song, which is produced in most cicadas when disturbed or handled. These signals are loud sounds that are widely thought to deter predators (Claridge 1985, Fonseca 1991). Both calling song and courtship songs can last without interruption for many minutes while alarm sounds can last only for one or two seconds.

##### Calling song.

**Figure 5–7. F7:**
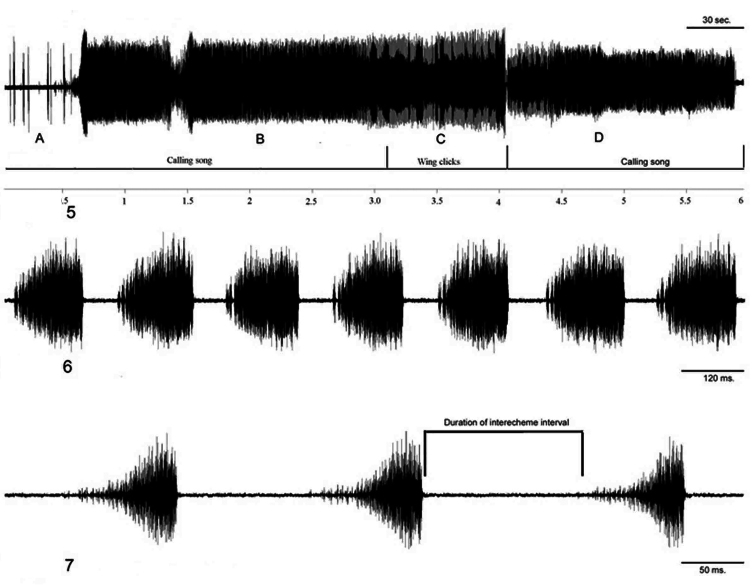
**5** *Cicadatra platyptera*, example of a male song, a whole song **A** Beginning of calling song **B** Calling song **C** Courtship song **D** Calling song (scale= 30 second) **6** *Cicadatra platyptera*, male song, 7 echemes from calling song (scale=120 ms) 7 *Cicadatra platyptera*, male song, 3 echemes from calling song (scale=50 ms).

The phrases of this song are produced by the timbals (Fig. 5B, D). The calling song consists of echeme sequences and intervals between echemes. Echeme duration is in average 122.7 (50–188) ms and interval duration between echemes average 91.2 (40–213) ms (Figs 6, 7). Echeme duration of the calling song if produced after the courtship song (Fig. 5D) is slightly different, in average 133 (75–277) ms and intervals between echemes are in average 80 (36–212) ms long. Calling song sometimes starts with irregular echemes lasting 5–15 ms (Fig. 5A).

##### Frequency range.

The spectrum of these acoustic signals includes frequencies from about 5.5 to 12 kHz with a maximum 6 kHz, 8 kHz and 10 kHz (Fig. 18).

##### Courtship song.

**Figure 8–10. F8:**
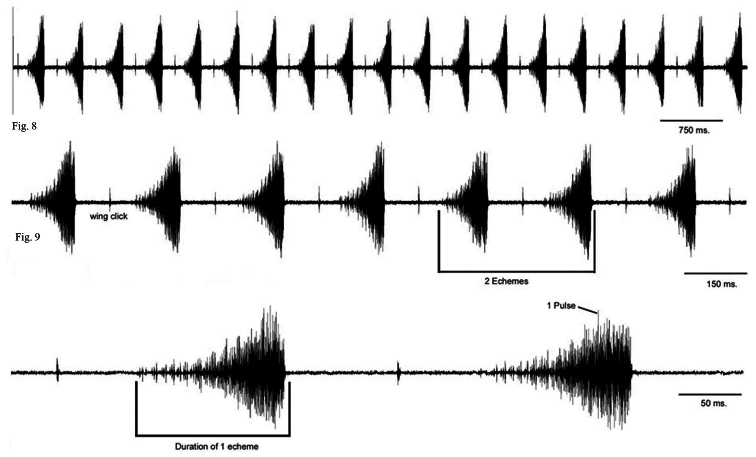
**8** *Cicadatra platyptera*, male song, 19 echemes from courtship song (scale=750 ms) **9** *Cicadatra platyptera*, male song, 7 echemes from courtship song (scale=150 ms) **10** *Cicadatra platyptera*, male song, 2 echemes from courtship song (scale=50 ms).

The phrases of this song are produced by the timbals and fore wings in succession. This song develops from the calling song, lasts for some minutes and an individual can continue with another sequence of calling song. The calling song consists of an echeme sequence, and wing clicks in the middle of the intervals between echemes (Figs 8–10). Each echeme averages 104 (83–132) ms and interval durations between echemes average 132 (48–176) ms. The duration of the courtship song is about one or a few minutes (Figs 5C, 8, 9, 10).

##### Frequency range.

The spectrum of these acoustic signals includes frequencies from about 5.5 to 12 kHz with a maximum between 6 kHz and 6.5 kHz (Fig. 19). The courtship song wing clicks, amplitude spectra showing audible frequencies ranging from about 1.7–4.6 kHz and with a maximum 3 kHz (Fig. 20).

##### Alarm sounds.

**Figure 11–12.**
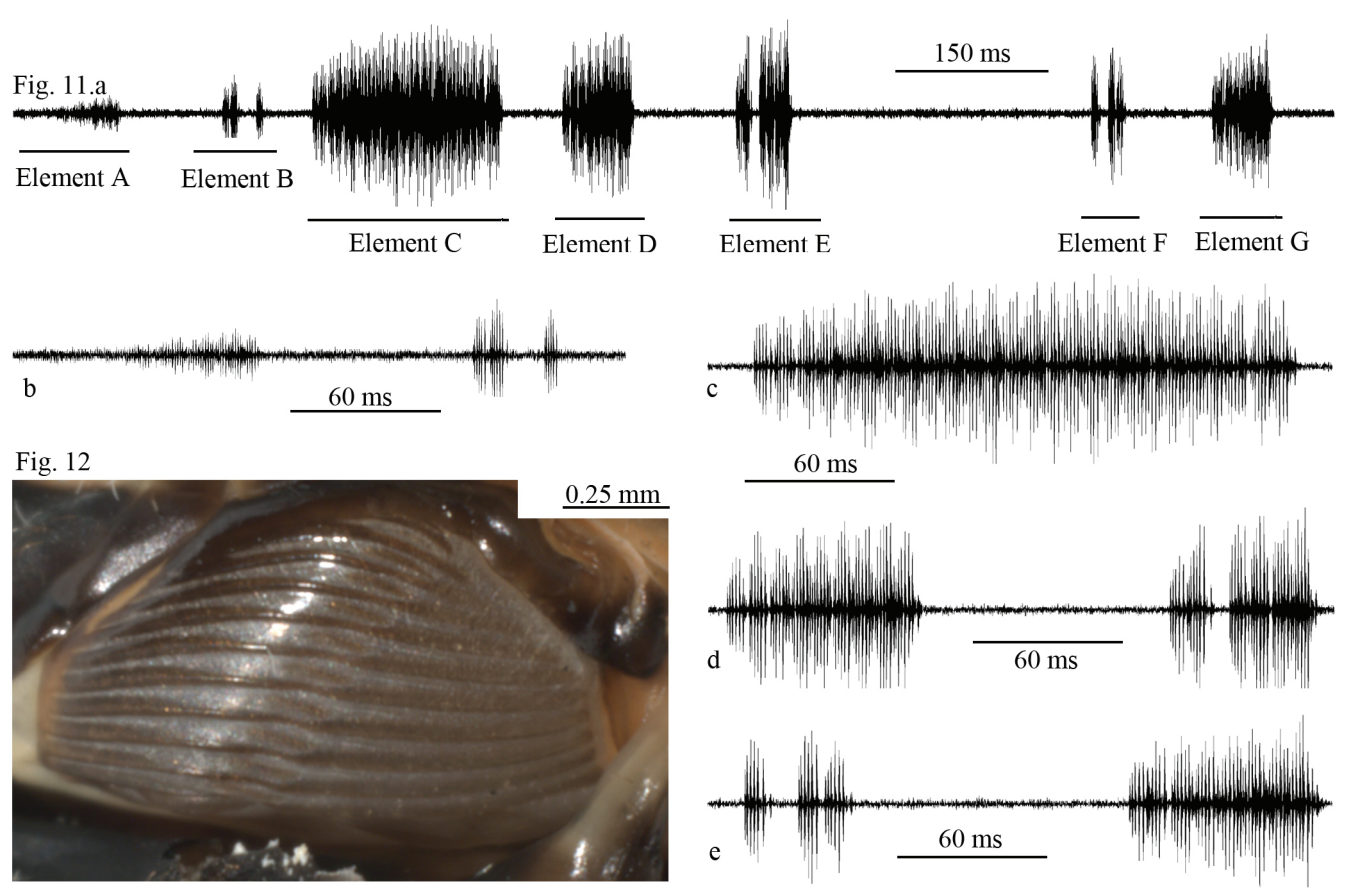
**11** *Cicadatra platyptera*, male alarm song, **a** 1 phrase (scale=150 ms) **b** element **A** and **B** (scale = 60 ms) **c** element **C** (scale=60 ms) **d** element **D** and **E** (scale=60 ms) **e** element **F** and **G** (scale = 60 ms) **12***Cicadatra platyptera*, right male timbal (scale=0.25 mm).

When the animals are disturbed, they may produce these sounds. We evaluated a total of 6 recordings of alarm sounds of 6 different animals. The alarm sounds consist of irregular echemes in terms of duration, but generally one can find similar sound patterns or elements in different animals. The alarm song lasts 1300–1350 ms, and consists of 7 elements (Fig. 11A). Elements A last 74–81 ms, elements B last 46–50 ms (Fig. 11B), elements C last 206–210 ms (Fig. 11C), elements D last 77–80 ms, elements E last 60–62 ms (Fig. 11D), elements F last 36–38 ms and elements G last 36–38 ms (Fig. 11E). The interval between elements A and B is 104–107 ms, between B and C is 50–53 ms, between C and D is 63–64 ms, between D and E is 107–109 ms, between E and F is 319–323 ms and the interval between elements F and G is 91–94 ms.

##### Frequency range.

The spectrum of these acoustic signals includesfrequencies from about 5.5 to 12 kHz with a maximum about 8 kHz (Fig. 21).

##### Examined material for *Cicadatra platyptera*.

Corum, Sungurlu, Kemallı village, 14.7.2006, 790 m, 2 ♂, 40°08'269"N; 34°31'303"E (temperature 33°C); Central district, Mecitozu, Uçkoy, Simalı district, 13.7.2004, 815 m, 1 ♂, 40°21'438'N; 35°08'628"E; Central district, Beydili-Catak fork, 13.7.2011, 865 m, 6♂, (temperature 30°C), 40°36'549"N; 34°54'075"E; Çorum-Cemilbey way, Alike Vineyard, 900 m, 15.7.2012, 2 ♂; Central district, Karsıyaka street, 900 m, 2.8.2011, 2 ♂, 40°32'937"N; 34°59'137"E (temperature 33.6°C), (Fig. 17).

##### Distribution for *Cicadatra platyptera*.

Europe, North Africa, Russia, Turkey, Iran, Israel, Lebanon, Syria (Dlabola 1974, Lodos and Kalkandelen 1981, Ahmed and Sanborn 2010).

##### Recorded distribution in Turkey for *Cicadatra platyptera*.

Izmir: Bornova–Ilica-Karaburun; Giresun, Sebinkarahisar; Gumushane: Torul; Kahramanmaras, Goksun; Konya: Eregli; Mugla: Bodrum; Sivas: Hafik, (Lodos and Kalkandelen 1981, Boulard 1995), (Fig. 17).

**Table 1.** Comparison of morphological characters of two *Cicadatra* species.

**Characters (for males)\Species (mm)**		***Cicadatra platyptera***	***Cicadatra atra***
Body length with wings	Range	26–30	22.8–26
	m±sd	27.83±1.72	24.96±0.84
	N	6	6
Body length	Range	22–24	18–21
	m±sd	22.83±0.75	18.83±1.17
	N	6	6
Head length	Range	1.19–1.65	1.03–1.56
	m±sd	1.43±0.20	
	N		5 1.28±0.25
Vertex length	Range	0.76–1.10	0.73–1.07
	m±sd	0.99±0.19	0.86±0.16
	N		5
Transverse grooves number	Range	12–14	10–12
	m±sd	12.5±0.6	11±1
	N	63	5
Eye diameter	Range	1.81–1.99	1.60–1.72
	m±sd	1.90±0.03	1.67±0.05
	N	6	5
Distance between eyes	Range	2.53–3.60	2.73–3.25
	m±sd	3.11±0.62	2.95±0.21
	N	6	5
Distance between lateralocelli	Range	0.73–1.06	0.77–1.02
	m±sd	0,90±0,22	0.86±0.09
	N	6	
Pronotum length	Range	2.00–2.53	2.08–2.70
	m±sd	2.38±0.20	2.35±0.22
	N	6	6
Minimum width of pronotum	Range	4.70–6.33	4.92–6.17
	m±sd	5.68±0.60	5.44±0.43
	N	6	6
Maximum width of pronotum	Range	6.01–8.20	6.20–8.01
	m±sd	7.40±1.54	6.84±0.67
	N		
Postclypeus length	Range	1.80–2.43	1.96–2.30
	m±sd	2.32±0.25	2.10±0.13
	N	65	
Rostrum length	Range	4.15–4.58	3.37–4.05
	m±sd	4.31±0.16	3.80±0.33
	N	6	5
Mentum length	Range	1.20–1.35	1.28–1.40
	m±sd	1.29±0.01	1.34±0.05
	N	6	5
Labium length	Range	2.87–3.21	2.2–2.69
	m±sd	3.00±0.2	2.54±0.20
	N	6	5
Mesonotum length	Range	4.62–5.35	3.85–4.88
	m±sd	4.96±0.30	4.44±0.58
	N	6	6
Maximum length of mesonotum	Range	6.40–7.21	5.12–6.70
	m±sd	6,73±0.43	5.95±0.65
	N	6	6
Fore femur length	Range	2.70–2.91	2.18–2.91
	m±sd	2.79±0.06	2.56±0.28
	N	6	5
Fore wing length	Range	21–24	18–22
	m±sd	22.60±0.70	20.02±1.48
	N	6	5
Fore wing width	Range	8.0–9.5	7.5–9.40
	m±sd	9.03±0.54	8.18±0.64
	N	6	6
Subcostal cell length	Range	2.40–2.73	1.76–2.11
	m±sd	2.53±0.10	1.92±0.15
	N	6	6
Operculum length	Range	4.32–4.91	2.97–4.72
	m±sd	4.48±0.07	3.42±0.65
	N	6	6
Operculum width	Range	2.77–4.79	3.39–4.46
	m±sd	4.01±0.28	3.84±0.35
	N	6	6
Apical width of operculum	Range	2.50–3.30	1.40–2.48
	m±sd	2.94±0.07	1.70±0.40
	N	6	6
Pygofer length	Range	3.16–3.40	2.93–3.70
	m±sd	3.43±0.54	3.29±0.25
	N	6	6
Dorsal beak length of pygofer	Range	0.76	0.50–0.80
	m±sd	0.73±0.045	0.65±0.15
	N		6
Aedeagus length	Range	2.25–2.5	**2**.25–2.56
	m±sd	2.38±0.18	2.44±0.16
	N	4	3
Sternite VIII length	Range	3.80–4.30	3.79–4.04
	m±sd	4.07±0.21	3.82±0.22
	N	6	6
Sternite VIII width	Range	2.60–2.88	2.17–2.60
	m±sd	2.70±0.17	2.33±0.20
	N	6	6

range - m- mean; sd- standard deviation; N- male number.

**Table 2. T2:** Acoustic parameters of *Cicadatra platyptera*.

**Locality**		**Duration echemes (ms)**	**Interval between echemes<br/> Duration**	**Maximum frequency**
Calling song	Range	50-188	40-213	5.5-12 kHz<br/> Maximum<br/> 6,8,10 kHz
	m	122.7±16.04	91.2±17.63	
	N	4	3	
	N	808	803	
Courtship song	Range	83-132	48-176	5.5-12 kHz<br/> Maximum<br/> 6-6.5 kHz<br/> For wing click<br/> 1.7-4.6 kHz<br/> Maximum 3 kHz
	m±d	104±6.33	132±10.9	
	N	3	3	
	n	238	238	
Calling song (after courtship song)	Range	75-277	36-212	5.5-12 kHz<br/> Maximum<br/> 6,8,10 kHz
	m±d	133±42.22	80±18.65	
	N	1	1	
	n	392	392	

Range - ; m- mean; sd- standard deviation; N- male number; n- sample number.

**Table 3. T3:** Alarm song parameters of *Cicadatra platyptera*.

**Element types**	**Parameters**	**Duration echemes (ms)**	**Interval between echemes<br/> Duration**
Element A	Range	74-81	104-107<br/> between elements A and B
	N	6	
	N	6	
Element B	Range	46-50	50-53<br/> between elements B and C
	N	6	
	N	6	
Element C	Range	206-210	63-64<br/> between elements C and D
	N	6	
	N	6	
Element D	Range	77-80	107-109<br/> between elements D and E
	N	6	
	n	6	
Element E	Range	60-62	319-323<br/> between elements E and F
	N	6	
	n	6	
Element F	Range	36-38	91-94<br/> between elements F and G
	N	6	
	n	6	
Element G	Range	36-38	
	N	6	
	n	6	

**Table 4.** Comparison of mean body measurements of *Cicadatra atra* and *Cicadatra platyptera*.

	**Species**	**N**	**Mean**	**Std. Error**	**Df**	**T**	Sig.*
**Body length with wings**	*Cicadatra atra*	6	24.967	0.543	10	-3.228	**0.009**
	*Cicadatra platyptera*	6	27.833	0.703			
Body length	*Cicadatra atra*	6	18.833	0.477	10	-0.188	0.854
	*Cicadatra platyptera*	6	19.500	3.510			
Head length	*Cicadatra atra*	5	1.284	0.113	9	-1.204	0.259
	*Cicadatra platyptera*	6	1.435	0.067			
Vertex length	*Cicadatra atra*	5	0.858	0.073	9	-1.475	0.174
	*Cicadatra platyptera*	6	0.997	0.061			
Transverse groove number	*Cicadatra atra*	5	11.000	0.447	9	0.000	1.000
	*Cicadatra platyptera*	6	11.000	2.017			
**Eye diameter**	*Cicadatra atra*	5	1.674	0.023	9	-6.365	**0.000**
	*Cicadatra platyptera*	6	1.902	0.026			
Eyes between length	*Cicadatra atra*	5	2.948	0.095	9	-0.887	0.398
	*Cicadatra platyptera*	6	3.113	0.150			
Distance between lateral ocelli	*Cicadatra atra*	5	0.862	0.043	9	-0.928	0.377
	*Cicadatra platyptera*	6	0.925	0.050			
Pronotum length	*Cicadatra atra*	6	2.348	0.090	10	-0.271	0.792
	*Cicadatra platyptera*	6	2.382	0.084			
Minimum pronotum width	*Cicadatra atra*	6	5.445	0.178	10	-0.805	0.440
	*Cicadatra platyptera*	6	5.688	0.244			
Maximum pronotum width	*Cicadatra atra*	6	6.842	0.274	10	-1.238	0.244
	*Cicadatra platyptera*	6	7.395	0.353			
Postclypeus length	*Cicadatra atra*	5	2.102	0.059	9	-1.113	0.295
	*Cicadatra platyptera*	6	2.315	0.166			
**Rostrum length**	*Cicadatra atra*	5	3.790	0.150	5.467	-3.201	**0.021**
	*Cicadatra platyptera*	6	4.313	0.065			
Mentum length	*Cicadatra atra*	5	1.284	0.067	9	-0.015	0.988
	*Cicadatra platyptera*	6	1.285	0.025			
**Labium length**	*Cicadatra atra*	5	2.536	0.089	9	-4.821	**0.001**
	*Cicadatra platyptera*	6	3.002	0.049			
Minimum length of mesonotum	*Cicadatra atra*	6	4.437	0.239	10	-1.987	0.075
	*Cicadatra platyptera*	6	4.965	0.117			
**Maximum length of mesonotum**	*Cicadatra atra*	6	5.945	0.266	10	-2.554	**0.029**
	*Cicadatra platyptera*	6	6.732	0.156			
Fore femur length	*Cicadatra atra*	5	2.562	0.127	9	-1.863	0.095
	*Cicadatra platyptera*	6	2.792	0.040			
**Fore wing length**	*Cicadatra atra*	5	20.200	0.663	9	-3.048	**0.014**
	*Cicadatra platyptera*	6	22.583	0.455			
**Fore wing width**	*Cicadatra atra*	6	8.183	0.261	10	-2.365	**0.040**
	*Cicadatra platyptera*	6	9.025	0.242			
**Subcostal cell length**	*Cicadatra atra*	6	1.918	0.063	10	-7.006	**0.000**
	*Cicadatra platyptera*	6	2.530	0.060			
**Operculum length**	*Cicadatra atra*	6	3.420	0.267	10	-3.781	**0.004**
	*Cicadatra platyptera*	6	4.483	0.089			
Operculum width	*Cicadatra atra*	6	3.827	0.145	10	-0.576	0.577
	*Cicadatra platyptera*	6	4.010	0.283			
**Apical width of operculum**	*Cicadatra atra*	6	1.702	0.165	10	-5.785	**0.000**
	*Cicadatra platyptera*	6	2.943	0.137			
Pygofer length	*Cicadatra atra*	6	3.287	0.102	10	-0.795	0.445
	*Cicadatra platyptera*	6	3.432	0.151			
Dorsal beak length	*Cicadatra atra*	6	0.655	0.043	9	-1.153	0.279
	*Cicadatra platyptera*	5	0.750	0.074			
Aedeagus length	*Cicadatra atra*	3	2.437	0.095	5	0.442	0.677
	*Cicadatra platyptera*	4	2.388	0.066			
Sternite VIII length	*Cicadatra atra*	6	3.823	0.089	10	-2.103	0.062
	*Cicadatra platyptera*	6	4.068	0.076			
**Sternite VIII width**	*Cicadatra atra*	6	2.328	0.079	10	-3.912	**0.003**
	*Cicadatra platyptera*	6	2.703	0.055			

* Differences are significant P< 0.05. Diagnosischaractersare shownwith bold words and numbers.

#### Cicadatra atra

(Olivier, 1790)

http://species-id.net/wiki/Cicadatra_atra

**Figure 13.**
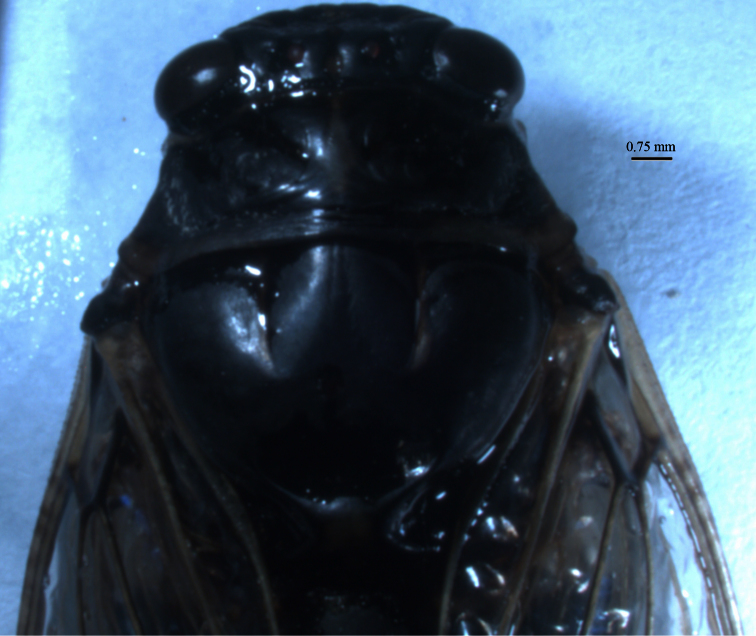
*Cicadatra atra*, head + pronotum+ mesonotum (scale= 0.75 mm).

**Figure 14.**
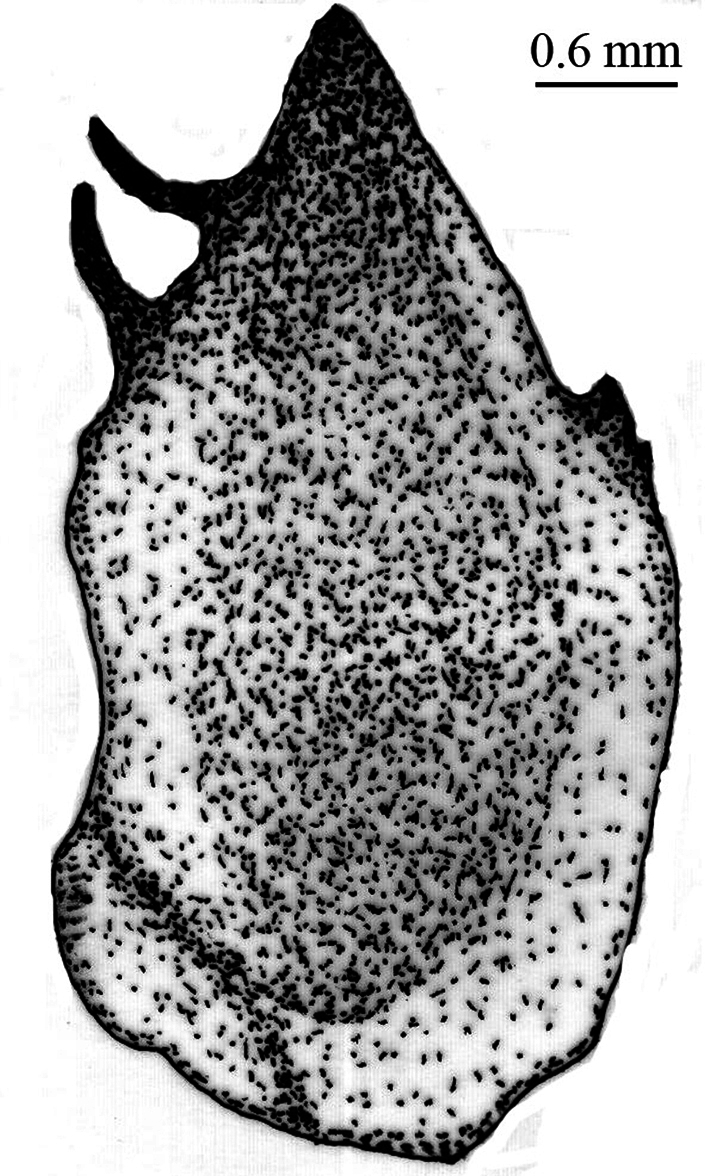
*Cicadatra atra*, pygofer (scale= 0.6 mm).

**Figure 15.**
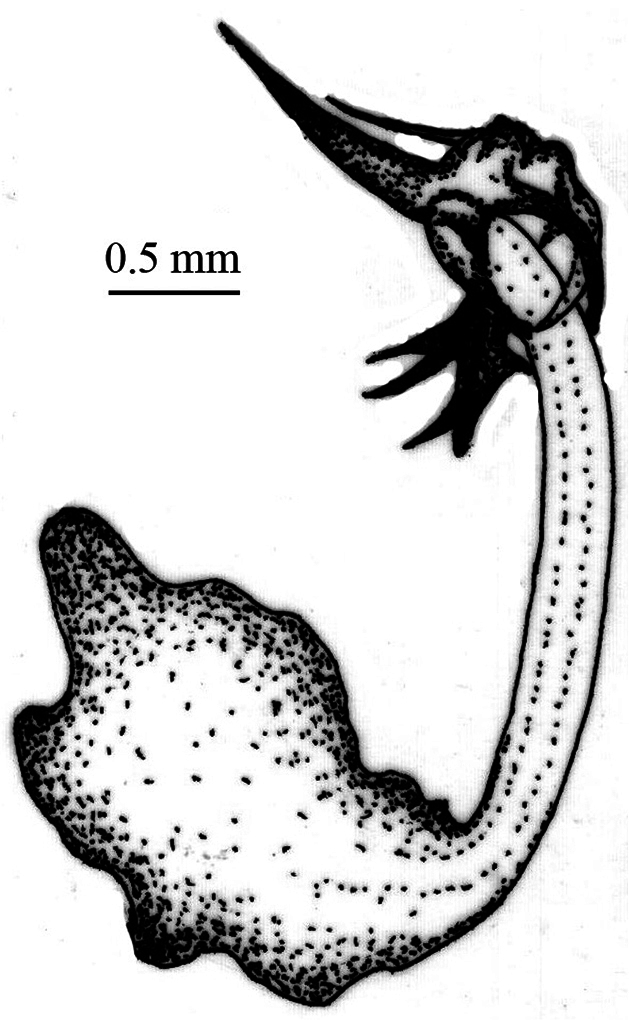
*Cicadatra atra*, aedeagus (scale=0.5 mm).

**Figure 16.**
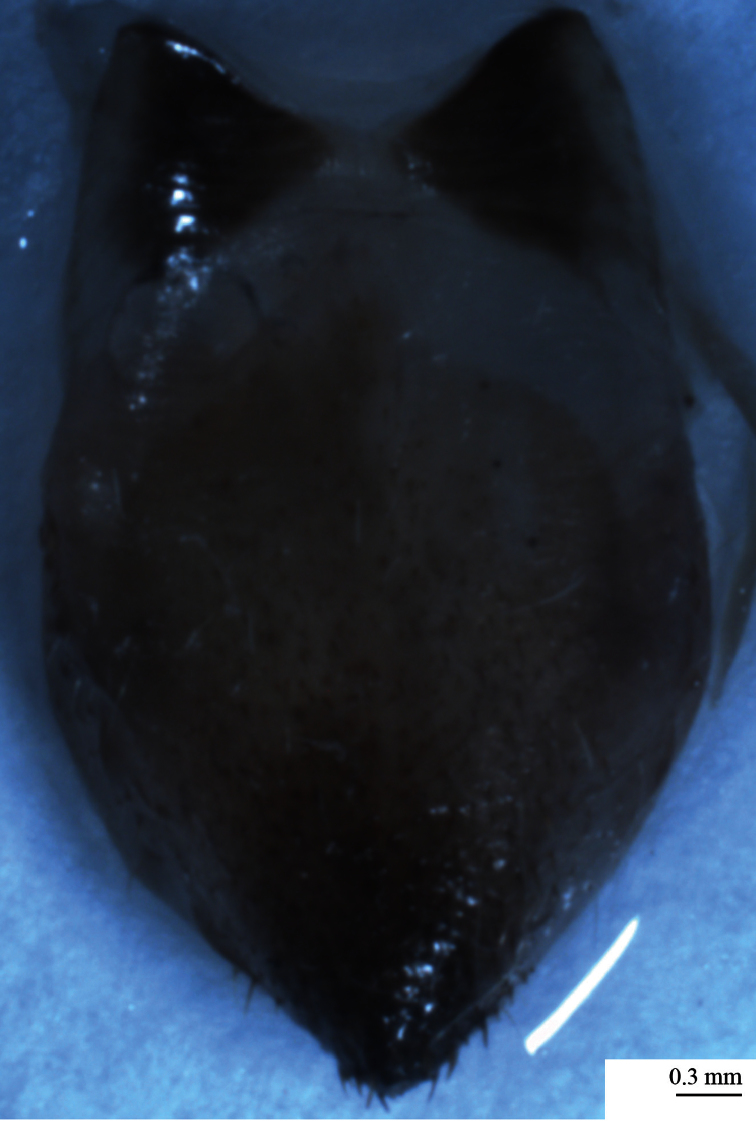
*Cicadatra atra*, sternite VIII (scale= 0.3 mm).

**Figure 17.**
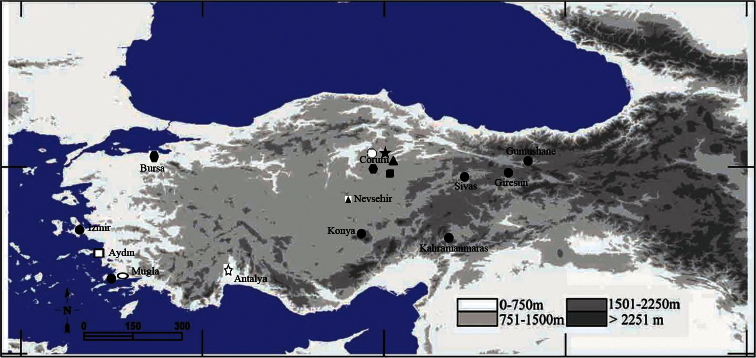
Map of Turkey, filled symbols represent localities of *Cicadatra platyptera* and empty shapes represent localitiesof *Cicadatra atra*.

Figs 13 –17 Table 1 , 4

##### Examined material.

Antalya: Korkuteli, Ziyarettepe, 3.7.2003, 1 ♂; Aydin, Kusadasi, Guzelcamlı, 26.6.2011, 1 ♂; Bursa: Iznik, Omerli vineyards, 7.8.2005, 1 ♂; Corum: Central district, Eskikoy village, 650 m, 8.7.2011, 1 ♂; Mugla, Bodrum, Aspat, 18.7.2009, 1 ♂; Nevsehir: Gulsehri-Hacıbektas way, 10.km, 1300 m, 22.7.2012, 1 ♂, (Fig. 17).

##### Distribution.

France incl. Corsica; Spain; Italy incl. Calabria, Sicily; Cyprus; Albania; Greece; Georgia; former Southern U.S.S.R.; Macedonia,; Serbia, Slovenia, Croatia; Czechoslovakia; Turkey; Iran (Duffels and Laan 1985; Mozaffarian and Sanborn 2010, Onder et al. 2011 etc.).

##### Recorded distribution in Turkey for *Cicadatra atra*.

Adana, Amasya, Ankara, Antalya, Bitlis, Edirne, Erzincan, Gaziantep, Istanbul, Izmir, Kahramanmaras, Kayseri, Mugla, Sivas, Siirt, Sanlıurfa (Onder et al. 2011).

## Discussion and conclusion

The shape, color, structure of body and genital structure of *Cicadatra platyptera* specimens collected in Turkey were examined in detail. The features of these specimens were compared with the descriptions given for this taxon by Schedl (1999) and Gogala (2013), and closely matched them. In addition in this study, measurements of external morphological structures with statistical analyses not given by Schedl (1999) or Gogala (2013) are presented with other morphological characters.

The songs produced by the male *Cicadatra platyptera* consist of two repeated phrases; calling song and courtship song. In this study, we also recorded and evaluated the alarm song of *Cicadatra platyptera*. At the beginning, first two songs are collectively repeated songs of *Cicadatra platyptera*, and they continue for some minutes. There are similar sound producing mechanisms in other species of *Cicadatra* (Gogala and Trilar 1998, Gogala 2013). In the study by Gogala et al. (2005), only basic information was given about the courtship song; previous studies did not report the irregular echemes at the beginning of calling songs. We recorded the irregular echemes which last for 5–15 ms at the beginning of calling songs.

In this study, it was determined that courtship song data of the examined samples were similar to the courtship song data reported for this taxon by Gogala (2013) and Gogala et al. (2005). According to our data, echeme durations in the calling song are longer than echeme durations in the courtship song, but interval durations between echemes in the courtship song take longer than the interval durations between echemes in the calling song. As a result, echeme duration plus interval between echeme is longer in the courtship song than the calling song.

The alarm song is different from the calling song. Fonseca (1991) stated that the alarm songs of *Tettigettalna argentata* (Olivier, 1790), *Tettigettalna estrellae* Boulard, 1982, and *Tettigettalna josei* Boulard, 1982 are different from each other. In this study, we only recorded the alarm song of *Cicadatra platyptera* therefore, whether the alarm song of *Cicadatra* Kolenati, 1857 is taxonomically significant or not will be revealed when the songs of other species are evaluated.

The frequency of the courtship song’s wing clicks (ranges from 1.7-4.6 kHz and with a maximum 3 kHz) is lower than that of the calling and courtship song’s echeme (ranges from 8.8 to 12 kHz and with a maximum 6, 8, and 10 kHz).

A continuous song is absent in *Cicadatra platyptera* (in *Cicadatra atra*, continuous song present), in both species wing clicks follow short timbale echemes but the repetition rate is about two times higher in *Cicadatra platyptera*. To improve our knowledge on singing cicadas of Turkey, data from various foreign collections should be included and more field work with the use of bioacoustics methods should be done in the future.

*Cicadatra platyptera* is related to *Cicadatra atra* (Olivier, 1790) but there are differences between *Cicadatra platyptera* and *Cicadatra atra* as reported in previous studies (Boulard 1995, Schedl 1999, Gogala 2013) and the examined material from Turkey. Some diagnostic characters are: generally body color yellowish with black patterns (in *Cicadatra atra*, generally body color is blackish brown, Fig. 13), mesonotum has yellowish pattern in the middle (in *Cicadatra atra*, mesonotum has usually blackish pattern in the middle), pygofer has sparsely distributed setae on apical and basal part (in *Cicadatra atra*, pygofer has no setae, Fig. 14), upper lobes of pygofer are nearer to the dorsal beak of pygofer (in *Cicadatra atra*, upper lobes of pygofer are farther from the dorsal beak of pygofer). The edges between dorsal beak and upper lobes of pygofer are like a half moon (in *Cicadatra atra*, edges between dorsal beak and upper lobes of pygofer are straigth), apex of the sternite VIII widened (in *Cicadatra atra*, apex of the sternite VIII acute, Fig. 16). In terms of statistical analyses, differences in mean body length, eye length, rostrum length, labium length, maximum width of the mesonotum, fore wing length, fore wing width, subcostal cell length, operculum length, apical width of operculum length, sternite VIII width of *Cicadatra atra* and *Cicadatra platyptera* were significant (Table 4).

**Figure 18. F15:**
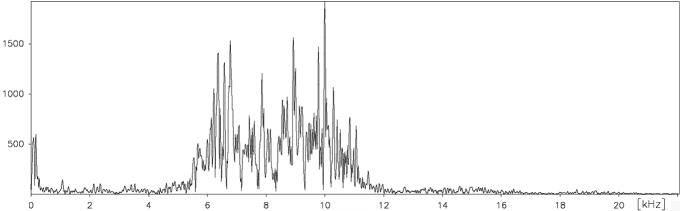
Frequency pattern of a typical calling song echeme produced by *Cicadatra platyptera*.

**Figure 19. F16:**
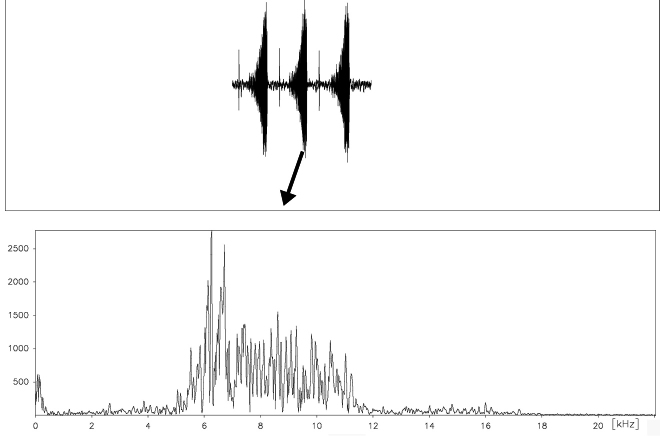
Frequency pattern of a typical courtship song echeme produced by *Cicadatra platyptera*.

**Figure 20.**
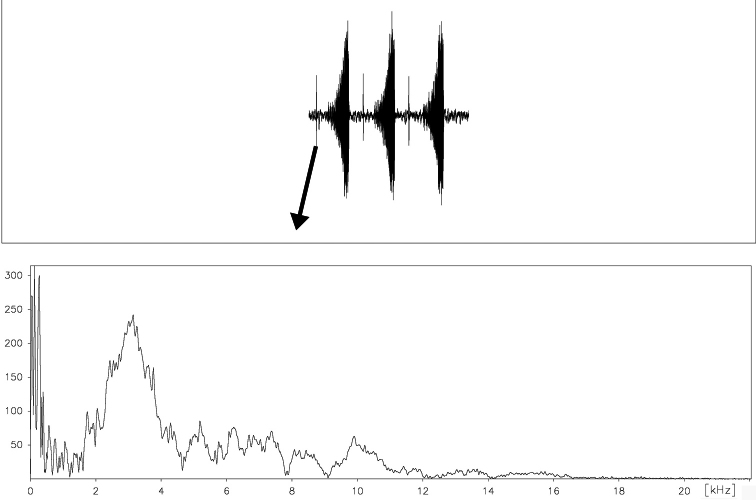
Frequency pattern of a typical courtship song’s wing clicks echeme produced by *Cicadatra platyptera*.

**Figure 21.**
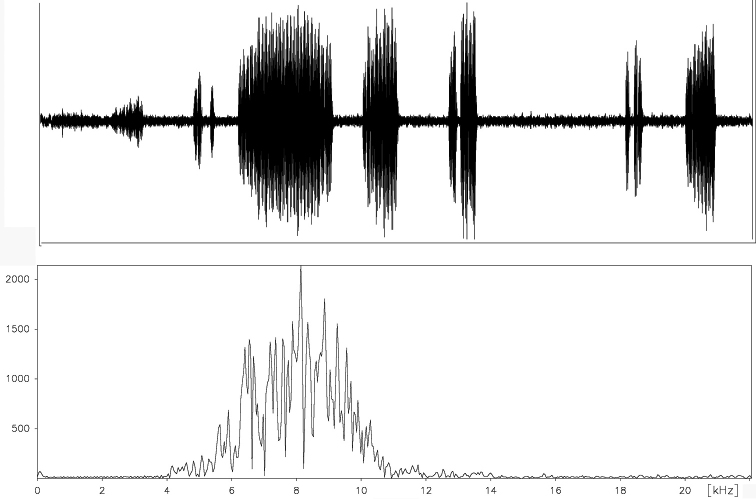
Frequency pattern of a typical C elements of alarm song produced by *Cicadatra platyptera*.

## Supplementary Material

XML Treatment for Cicadatra platyptera

XML Treatment for Cicadatra atra
